# For Some Sensory Neurons, Motor Response Shapes Their Output

**DOI:** 10.1371/journal.pbio.0040429

**Published:** 2006-11-28

**Authors:** Richard Robinson

Among the biggest surprises in the early days of neurobiology was the discovery that some sensory neurons responded most when a stimulus was presented just so. When a bar in the visual field, for instance, was oriented not too much this way, not too much that way, but with exactly the right inclination, the neuron’s firing rate was maximum and tailed off in either direction. Such single-peaked responses are well understood as an efficient means to encode information about orientation and other features—whether visual, auditory, or tactile.

But single-peaked response curves are not the only kind. In fact, monotonic curves—those which steadily increase or decrease without a middle peak—are ubiquitous among sensory neurons. They are especially common in the somatosensory system, which includes those sensors that tell us what is touching our skin and how our body is moving and that provide vital information for controlling muscles. For many of these neurons, more stimulus means more response.

A long-standing question in theoretical neurobiology is what general conditions promote monotonic, rather than single-peaked, response curves? In a new study, Emilio Salinas provides evidence that monotonic sensory inputs are favored when monotonic motor outputs are desired. More generally, he shows that the shapes of the optimal sensory response curves should be matched to the type of the motor output that they are linked to.

The author began by constructing the simplest of mathematical models, in which a small number of sensory neurons are directly linked to a larger number of motor neurons (in complex nervous systems such as ours, several layers of intervening neurons mediate between the two). He asked what kind of sensory responses would be needed to drive the desired output of the nervous system, such as a monotonic motor response. The model provided several possible answers, including single-peaked sensory curves. However, the most efficient type of sensory activity—the one that achieved the highest accuracy and highest tolerance to noise from the fewest neurons—was one where the sensory responses were also monotonic.

**Figure:**
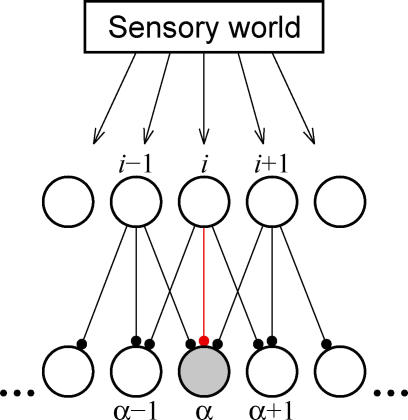
The author describes a computational model in which sensory information is received by one set of neurons (*i*) and transferred to another set (α), which control behavior.

This model provides insight into several heretofore unexplained observations. Binocular vision allows the two eyes to be focused using information about the disparity between the current focal length and the desired one. When the disparity is great, a relatively large and stereotyped oculomotor response is required to quickly achieve focus; when the disparity is small, the behavioral response is less constrained, so a larger variety of eye movements is possible. Several different kinds of visual processing neurons that respond to binocular disparity have been discovered and are generated by the model as well. One kind, which includes neurons that respond monotonically to large disparities, most likely helps to drive the large refocusing response.

The author’s linkage of motor output to sensory curve shape also helps explain a curious aspect of single-peaked response curves, namely their variety of possible widths. A narrow curve is produced by a neuron tightly tuned to its ideal stimulus—it fires very little when the stimulus varies even slightly from the ideal. A broad curve, on the other hand, indicates a neuron that is responsive to a wider range of inputs. Salinas shows that this variation is expected when the motor response must vary. The bat, for instance, must maneuver faster as it gets closer to its prey, and studies have shown that its echolocation system includes both broadly and narrowly tuned auditory sensors, which are most active when the prey is far or near, respectively. The author proposes that the broader maneuvers of the bat far from its prey are driven more by the more broadly tuned sensors, while the more rapid motor responses as it closes in are linked with the narrowly tuned sensors. Initial data from animal experiments support this model.

Linking the shape of the sensory response curves to the motor actions that may follow a stimulus provides a theoretical framework for the existence of monotonic response curves. This may help explain the wide diversity of sensory curves seen in a variety of situations, including collision avoidance, orienting responses, evasive maneuvers, and other well-defined sensorimotor behaviors.

